# 224. Immunosuppression and RSV Prefusion F Protein Vaccine Effectiveness in Older Adults: A Prespecified Subanalysis from the DAN-RSV Trial

**DOI:** 10.1093/ofid/ofaf695.007

**Published:** 2026-01-11

**Authors:** Anne Marie Reimer Jensen, Mats C H Lassen, Niklas D Johansen, Sine H Christensen, Negar Aliabadi, Kristoffer Skaarup, Daniel Modin, Kira Janstrup, Brian L Claggett, Carsten S Larsen, Lykke Larsen, Lothar Wiese, Matias G Lindholm, Michael Dalager-Pedersen, Maria Dons, Katrine Bernholm, Filip S Davidovski, Lisa S Duus, Camilla I Ottosen, Anne B Nielsen, Julie Borchsenius, Caroline Espersen, Güldas Köse, Frederik Fussing, Lars Køber, Scott D Solomon, Jens Ulrik Jensen, Cyril Jean-Marie Martel, Bradford D Gessner, Claudia Schwarz, Elisa Gonzalez, Mette Skovdal, Pingping Zhang, Elizabeth Begier, Tor Biering-Sørensen

**Affiliations:** Copenhagen University Hospital - Herlev and Gentofte, Copenhagen, Hovedstaden, Denmark; Gentofte University Hospital, Copenhagen, Hovedstaden, Denmark; Copenhagen University Hospital - Herlev and Gentofte, Copenhagen, Denmark, Copenhagen, Hovedstaden, Denmark; Gentofte University Hospital, Copenhagen, Hovedstaden, Denmark; Pfizer, New York, NY; Gentofte University Hospital, Copenhagen, Hovedstaden, Denmark; Copenhagen University Hospital - Herlev and Gentofte, Copenhagen, Denmark, Copenhagen, Hovedstaden, Denmark; Copenhagen University Hospital - Herlev and Gentofte, Copenhagen, Denmark, Copenhagen, Hovedstaden, Denmark; Brigham and Women’s Hospital, Harvard Medical School, Boston, MA, USA, Boston, Massachusetts; Aarhus University Hospital, Aarhus, Midtjylland, Denmark; Odense University Hospital, Odense, Syddanmark, Denmark; Roskilde Hospital, Roskilde, Sjelland, Denmark; Zealand University Hospital, Copenhagen, Hovedstaden, Denmark; Aalborg University Hospital, Aalborg, Nordjylland, Denmark; Gentofte University Hospital, Copenhagen, Hovedstaden, Denmark; Gentofte University Hospital, Copenhagen, Hovedstaden, Denmark; Gentofte University Hospital, Copenhagen, Hovedstaden, Denmark; Copenhagen University Hospital - Herlev and Gentofte, Copenhagen, Denmark, Copenhagen, Hovedstaden, Denmark; Gentofte University Hospital, Copenhagen, Hovedstaden, Denmark; Gentofte University Hospital, Copenhagen, Hovedstaden, Denmark; Gentofte University Hospital, Copenhagen, Hovedstaden, Denmark; Copenhagen University Hospital - Herlev and Gentofte, Copenhagen, Hovedstaden, Denmark; Gentofte University Hospital, Copenhagen, Hovedstaden, Denmark; Gentofte University Hospital, Copenhagen, Hovedstaden, Denmark; Copenhagen University Hospital – Rigshospitalet, Copenhagen, Hovedstaden, Denmark; Brigham and Women’s Hospital, Harvard Medical School, Boston, MA, USA, Boston, Massachusetts; Copenhagen University Hospital - Herlev and Gentofte, Copenhagen, Denmark, Copenhagen, Hovedstaden, Denmark; SSI, Copenhagen, Hovedstaden, Denmark; EpiVac Consulting, Anchorage, Alaska; RWE Platform, Vienna, Wien, Austria; Pfizer Vaccines, Collegeville, Pennsylvania; Pfizer Denmark, Ballerup, Hovedstaden, Denmark; Pfizer Vaccines, Collegeville, Pennsylvania; Pfizer Vaccines, Collegeville, Pennsylvania; Department of Cardiology, Herlev and Gentofte Hospital, Copenhagen, Hovedstaden, Denmark

## Abstract

**Background:**

Immunosuppressed (IS) individuals are at high risk of severe respiratory syncytial virus (RSV) complications. This prespecified analysis of DAN-RSV evaluated the effectiveness of a bivalent RSV prefusion F protein-based vaccine (RSVpreF) in preventing RSV-related and all-cause cardio-respiratory hospitalizations among individuals with and without IS.

**Table 1.**
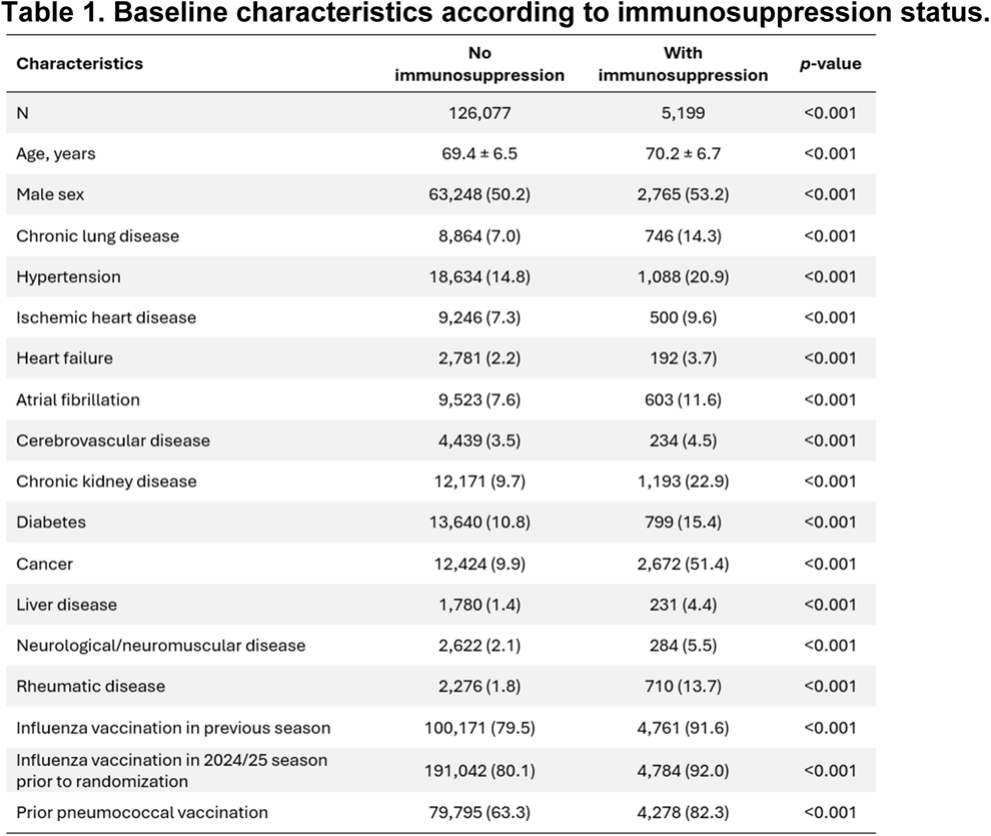
Baseline characteristics according to immunosuppression status. Values are presented as mean ± SD or n (%) with p-values from t-test or Pearson chi-squared test.

**Figure 1.**
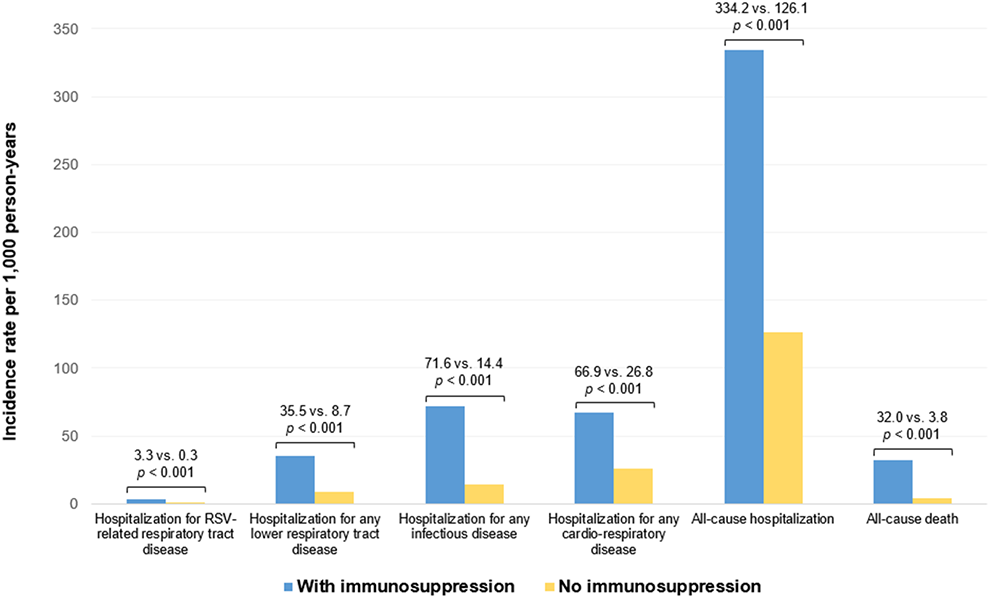
Hospitalization rates according to immunosuppression status in the overall study population. P-values from Poisson regression models.

**Methods:**

The DAN-RSV trial was a pragmatic, open-label randomized clinical trial conducted during the 2024/25 northern hemisphere winter. Adults aged ≥60 years were enrolled (Nov-Dec 2024) and randomized 1:1 to RSVpreF or no vaccine; only the RSVpreF arm were required to attend a study visit. Baseline and outcome data were obtained from nationwide registries. IS was defined using ICD10 or procedure codes and required ≥1 hospital encounter . Follow-up was from 14 days after vaccination/scheduled visit until May 31, 2025. The primary endpoint was hospitalization for RSV-related respiratory tract disease (RTD); secondary endpoints included hospitalizations for any respiratory and cardio-respiratory disease.

**Figure 2.**
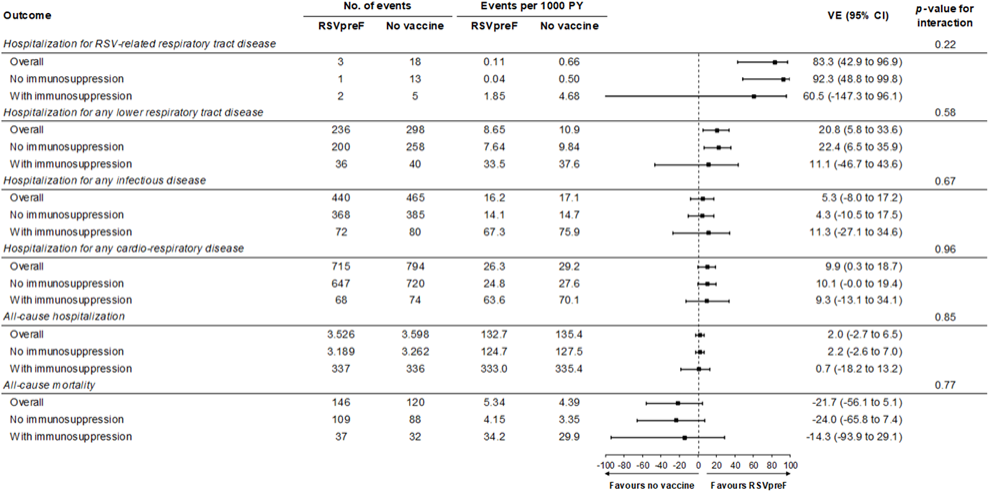
Vaccine effectiveness of RSVpreF compared with no vaccine according to immunosuppression status. Vaccine effectiveness was calculated as (1-incidence rate ratio*100%. Interaction p-values were estimated using a Poisson model with a sub-group-by-randomized treatment interaction term included. Abbreviations: PY = person-years; RSVpreF = respiratory syncytial virus prefusion F protein-based vaccine; VE = vaccine effectiveness.

**Results:**

Among 131,276 participants (65,642 RSVpreF; 65,634 no vaccine), 5,199 (4.0%) were IS. IS participants were older with more comorbidities (Table 1), as well as higher hospitalization rates (Fig. 1). Among the unvaccinated group, RSV-related RTD hospitalization rates were >9 times higher among IS persons (9.26; 4.96/0.5) (Fig. 2). Incidence of RSV-related RTD was lower in the RSVpreF group than in the no-vaccination group, with consistent effects regardless of baseline IS status (1.85 events vs. 4.68 events per 1000 person-years (PY); vaccine effectiveness (VE) 60.5% [95% CI: -147.3 to 96.1] in participants with IS compared with 0.04 events vs. 0.50 events per 1000 PY; VE 92.3% [95% CI: 48.8 to 99.8] in those without; *p*_interaction_ = 0.22). Consistent beneficial effects on hospitalization rates for were observed in the RSVpreF group vs no vaccine for other outcomes (*p* for interaction >0.05 for all).

**Conclusion:**

In adults ≥60 years, RSVpreF vaccination reduced hospitalizations for RSV-related cardio-respiratory disease, with consistent effects across immunosuppression subgroups. High rates of severe RSV related disease support the need for vaccination in this population.

**Disclosures:**

All Authors: No reported disclosures

